# Post-earthquake emotional and behavioral problems in children: parent experiences, coping strategies, and predictive factors

**DOI:** 10.3389/fpsyg.2025.1629996

**Published:** 2026-01-12

**Authors:** Yunus Tunç, Osman Tayyar Çelik, Cihangir Kaçmaz

**Affiliations:** 1 Vocational School of Health Services, Iğdir University, Iğdir, Türkiye; 2 Department of Child Development, Faculty of Health Sciences, Inönü University, Malatya, Türkiye; 3 Department of Child Development, Faculty of Health Sciences, Kayseri University, Kayseri, Türkiye

**Keywords:** child, disaster, earthquake, emotional and behavioral problems, parental warmth, psychological resilience

## Abstract

**Objective:**

In this study, the emotional and behavioral problems observed in children aged 6–11 years following the Kahramanmaras earthquakes in Türkiye on February 6, 2023, have been examined based on parental experiences, and it has been investigated whether the psychological resilience and warmth of the parents could predict these problems.

**Methods:**

The study was conducted using an explanatory sequential mixed-methods design. Fifty-five parents met the inclusion criteria for the qualitative phase, while 398 parents participated in the quantitative phase. Participants were identified using convenience and snowball sampling methods. Content analysis was applied to the qualitative data, while the quantitative data underwent analysis through descriptive statistics, T-tests, ANOVA, correlation studies, and hierarchical linear regression. In the quantitative phase, the Emotional and Behavioral Problems Questionnaire, the Brief Resilience Scale (BRS), and the Parent Acceptance-Rejection Questionnaire (PARQ) warmth/affection subscale were used.

**Results:**

The study revealed that, according to parents' perceptions, children's emotional problems after the earthquake revealed themselves with symptoms such as loneliness, constant anxiety about the earthquake, and fear of the dark, and parents reported various behavioral problems with their children. Parents tackled these issues by demonstrating resilience, warmth, protective actions, and trust-building. The quantitative research revealed substantial evidence of these issues' prevalence in children and the influence of parental factors and family dynamics.

**Conclusion:**

It was determined that parental psychological resilience is a key factor in predicting parents' reported emotional and behavioral problems for children, unlike parental warmth. Moreover, aspects such as household income and the number of siblings were also linked to parents' reported emotional and behavioral problems for children. The study thus provides a comprehensive framework for appreciating and aiding children's emotional and behavioral responses.

## 1 Introduction

Natural disasters represent major environmental events that lead to substantial material destruction and severely interrupt the functioning of communities, causing profound suffering and emotional distress for those affected (Bilik, 2023; Bonanno et al., 2010; Saeed and Gargano, 2022). Among these, earthquakes stand out for their abrupt onset and the potential for widespread devastation (Kurt and Gülbahçe, 2019; Sabuncuoglu et al., 2003). Such catastrophic events often lead to substantial property damage, loss of lives, bereavement over the loss of family and friends, limited access to essential resources and social services, displacement of populations, and significant interruptions in the normal functioning of societies. Moreover, it is acknowledged that children are among the most vulnerable to such natural disasters (Galvan et al., 2021; Sadeghloo and Mikhak, 2022). As global disaster events become more frequent and severe, children emerge as a particularly vulnerable group susceptible to their detrimental impacts. Their psychological vulnerability predisposes them to potential post-traumatic stress disorder (PTSD) or similar symptoms. Physically, they face heightened risks of mortality, injury, and illness (Sayili et al., 2024), and maltreatment (Seddighi et al., 2021). Disasters often disrupt or postpone their educational progress. It is crucial to acknowledge that children are not merely miniature adults. Their unique physical, cognitive, and social developmental stages render them disproportionately susceptible to the adverse impacts of disasters (McDonald-Harker et al., 2021; Reichert et al., 2023). Therefore, understanding children's unique vulnerabilities and adaptive capacities in the face of natural disasters, particularly earthquakes, is a critical turning point for examining the emotional and behavioral consequences that form the core of this study.

### 1.1 School-age children's post-earthquake reactions

Children's responses to earthquakes can vary depending on their developmental stage and the extent of loss experienced. One group best able to perceive earthquakes according to developmental characteristics is children in middle childhood, also known as the school-age period (Aral, 2023). Middle childhood is a critical developmental period in which children enter formal schooling and face increased cognitive, emotional, and social demands (Fernández-García et al., 2024). While positive experiences during this period can have long-term benefits, negative experiences can cause damage that may last for years in children. After an earthquake with devastating effects, children in this period have to struggle with many life stresses. In terms of social development, children may lose their social ties and become lonely by being separated from their peers after this disaster (Peek et al., 2018). Emotionally, problems such as anger outbursts, fear, anxiety, nightmares, sleep problems, anxiety, desire to be alone, and post-traumatic stress disorder can be seen (Kar and Bastia, 2006; Powell et al., 2021). Moreover, exposure to disasters can negatively impact students' academic performance by causing difficulties with focus, logical thought, drive, belief in their own abilities, and managing their effort (Mathew et al., 2021). Children in this period are better able to analyze their experiences due to the earthquake-related information they receive at school and their capability to perform concrete operations. However, during this period, children's responses to disasters can also be intense (Aral, 2023). Therefore, understanding the emotional and behavioral responses of school-aged children to earthquakes is crucial for identifying developmentally sensitive risk and protection models, which form the conceptual basis of this study.

### 1.2 Family processes: parental mental health, resilience, and warmth

Following disasters, children's social functioning deteriorates in the form of a decrease in academic achievement, breaks in social ties with peers, social withdrawal, reluctance to take responsibility, insecurity, and difficulty in establishing cooperation (Gibbs et al., 2021; Sapsağlam, 2019; Yorulmaz and Karadeniz, 2021). Damages and disruptions in a critically important environment such as school make the impairments in children's social functioning more evident (Pfefferbaum et al., 2015). Moreover, the quality of parent-child interaction has a multifaceted effect in either amplifying or mitigating the impacts of an earthquake on children. Indeed, disaster research has identified that the distress experienced by parents significantly predicts the symptoms of PTSD in children (Bountress et al., 2020; Chemtob et al., 2010). Therefore, parental mental health problems are a primary risk factor for the children who have survived earthquakes (Forresi et al., 2021). Additionally, the quality of parent-child interaction can influence children's recovery times and responses to the disaster aftermath. Hence, attention should be given to the psychological states and specific needs of parents, which can affect their well-being (North et al., 2018).

The resilience literature states that close relationships with caregivers, effective parenting of caregivers, and parents' resilience are important protective factors for children (Masten, 2021). In adults, resilience is defined as the capacity to maintain physical and psychological functionality in the face of intense adversities such as the loss of a loved one, exposure to violence, or life-threatening traumatic events (Bonanno et al., 2023). There are two primary aspects of psychological resilience. Firstly, adverse experiences contribute to the development of psychological resilience; secondly, this resilience builds resistance against potential future adversities, preparing the individual (Masten, 2014; Osofsky and Osofsky, 2018). An individual's psychological resilience can serve as a buffer against mental issues such as trauma, stress, and depression following death, disaster, and other devastating experiences. In this context, considering that parents' mental health post-disaster can influence their children's mental health (Powell and Leytham, 2014), parental psychological resilience can play a crucial role in helping children cope with negative experiences post-disaster. A secure parent-child relationship forms the basis for post-traumatic recovery and accelerates the process. Early intervention practices aim to reduce problems by improving this interaction, thereby facilitating the implementation of necessary programs, services, and resources (McDonald-Harker et al., 2021; Tosunoğlu and Seçer, 2025).

From an analytical perspective, parents' psychological resilience can be conceptualized as a protective factor at the individual level within Hobfoll's Conservation of Resources theory (COR), which represents the personal resources that help parents maintain emotional stability and effective caregiving in conditions of loss and threat (Hobfoll, 1989). Within this theoretical perspective, resilient parents can act as psychological “resource preservers,” reducing the transmission of trauma-related stress to children and thereby mitigating the development of emotional and behavioral problems.

Parental warmth is one of the factors that are effective in the psychological and emotional recovery of children after disasters. Parental warmth is considered an important psychological component that can effectively solve emotional and behavioral problems in children. Studies in the literature show that parental warmth is effective in helping children cope with stress, improving their general well-being, and has a protective effect on the recovery process after trauma (Gaylord-Harden et al., 2013; Rohner and Britner, 2002; Pinquart, 2017). In addition, parental warmth serves as a crucial protective factor that can mitigate symptoms of anxiety and depression among children (Butterfield et al., 2021). Finally, the literature also highlights parenting behaviors specifically warmth and responsiveness as key protective factors capable of buffering against these negative consequences (Riser et al., 2025).

Parental warmth is not only an emotional support after a disaster but also used as a coping strategy. Studies have shown that warm and compassionate parents' approaches help children cope with fear, anxiety, and loneliness (Marsac et al., 2013). In addition, it is reported that parents' compassion and warmth provide higher social functions in children and contribute to their high performance in academic achievement (Baker, 2017; Coley et al., 2011; Webster et al., 2013). Within the framework of COR theory, parental love can also be considered an interpersonal resource that helps protect and restore children's emotional security following the loss of resources caused by disasters (Hobfoll, 1989). As a result, it can be said that parental warmth plays an effective role in providing a safe environment in the family and contributing to children's well-being. The literature on whether parental warmth can predict the emotional and behavioral problems experienced by children after natural disasters is limited. As a result, addressing the relationship between parental warmth, an important aspect of parent-child interaction, and children's emotional and behavioral problems after the earthquake is important in contributing to the literature. Therefore, examining the relationship between parental warmth, an important aspect of parent-child interaction, and the emotional and behavioral problems of children after an earthquake contributes to the literature both theoretically and practically.

Intervention programs are necessary for children to quickly return to normalcy and adapt to new situations after an earthquake. Identifying which variables influence children's responses to disasters and their return to normalcy is important for determining risk groups and designing intervention programs (Pfefferbaum et al., 2015). Furthermore, the states of stress and anxiety of parents and their related behaviors can be a secondary source of stress for children. This relationship also prioritizes focusing on which characteristics and behaviors of parents can serve as a buffer against the adverse effects of disasters on children. Indeed, it is reported that the presence of supportive protective factors, such as parent and family support mechanisms, can balance the risk of negative psychological sequelae (Weems and Overstreet, 2008).

### 1.3 Theoretical framework: the conservation of resources

Understanding the emotional and behavioral problems seen in children after earthquakes is important for addressing resilience and coping with disasters in the context of community psychology (Bakic and Ajdukovic, 2019; Clissold et al., 2021). In this regard, Hobfoll's COR theory emphasizes that the actions of individuals to protect, use, and regain resources in case of stress have an impact on resilience (Hobfoll, 1989). Natural disasters such as earthquakes are reported to cause high stress by destroying the emotional, social, psychological, and material resources of individuals, and this loss of resources makes children more vulnerable (Sattler et al., 2018; Zhang et al., 2012). In addition, as a result of the loss of resources experienced by children, it is seen that the social support mechanisms and routines provided to them are disrupted, which causes a loss of trust in children (Platt et al., 2016).

(Norris and Kaniasty 1996), who dealt with the loss of social support after the disaster, examined the effect of this loss on the well-being of individuals, and (Cox and Perry 2011) examined the effect of re-establishment of social relations after the disaster on social resilience. These studies emphasize that the protection and regaining of social and individual resources emphasized in COR theory are related to resilience processes in children. Similarly, in their systematic study, (Cadamuro et al. 2021) found that maintaining children's social support networks and social ties contributed to children's resilience after disasters. Finally, (Scannell et al. 2016) stated that maintaining children's attachment to places such as home, school, or neighborhood provides a psychological resource function for children and contributes positively to their emotional development after a disaster. Within the framework of this literature, our study aims to contribute to the community psychology literature by examining the impact of resource losses and their recovery processes on children's social and emotional behaviors after disasters and to reveal the factors that predict emotional and behavioral problems in children.

### 1.4 Current study

On February 6, 2023, two major earthquakes with magnitudes of 7.8 Mw and 7.7 Mw, centered in the Pazarcik and Elbistan districts of Kahramanmaraş, and thousands of aftershocks occurred. These earthquakes caused extensive destruction in a wide area covering 11 provinces of Türkiye and Syria. Termed the “Disaster of the Century” by the World Health Organization, approximately 13.5 million people were affected by these earthquakes. Over 50,000 people died, more than 1.5 million were left homeless, and residents of these provinces were forced to relocate across the country. Most buildings were rendered unusable, and most regional hospitals became inoperable. Due to winter conditions and inaccessibility, serious health, shelter, and food problems arose in some areas (Güler Aksu and Imrek, 2023).

Türkiye is a developing country with a significant child population. In Türkiye, children constitute 26.9% of the population (Turkish Statistical Institute, 2022). Moreover, the number of children in the 11 provinces affected by the earthquake represents a considerable portion of the country's child population. Given Türkiye's susceptibility to earthquakes, it is imperative for the authorities to adopt adequate protective measures to minimize the behavioral, emotional, and psychological impacts of earthquakes on this vulnerable population. In this context, there is a need for comprehensive psychosocial support programs that not only address children's physical needs but also help them overcome the traumatic situations they may experience (Demir et al., 2010; Güler Aksu and Imrek, 2023).

In the aftermath of a disaster, a child's immediate environment is important in protecting them from harmful psychological effects (Ronan et al., 2008). Indeed, a comprehensive literature review by (Norris et al. 2002) indicates that the most significant risk factor for children is family factors. The impact of stress, anxiety, fear, and trauma observed in adults after a disaster on children is well-known. Parents can play a key role in minimizing the negative effects of disasters on children. More research is needed to focus on parental attitudes and behaviors that protect children in this context.

The literature on the impact of earthquakes on children mentions the importance of making an assessment both socially and individually. It is emphasized that the threats experienced by children after the earthquake, physical proximity, social support, and the parent-child relationship are affected by many factors (Norris et al., 2002; North et al., 2018; Pfefferbaum et al., 2015; Zhang et al., 2012). These factors play a role as both protective and risk factors for children. In particular, parents' perceptions of their post-traumatic experiences may play a role in overcoming these problems experienced by children (Raccanello et al., 2017; Yilancioglu and Özbaran, 2023). It is also known that loss of resources and decreased social support increase the negative psychological effects on children (Cadamuro et al., 2021). There are current criticisms about the reliability and validity of the data collected on children's experiences following disasters. As a matter of fact, (Demir et al. 2010) reported that children may experience a contraction in affect and a decrease in interest after a disaster, and thus they may have difficulty in expressing themselves. In this context, researchers emphasized the importance of collecting data from observers such as parents and teachers. In this context, it is suggested that parents' experiences can be used as a tool to understand both their own experiences and their perceptions of their children's experiences (van Wesel et al., 2014).

Although the psychological consequences of earthquakes on adults are relatively well documented, empirical studies focusing specifically on children's emotional and behavioral problems after disasters are limited in the Turkish context. Studies that bring together parental resilience and warmth as predictive factors affecting children's adjustment and recovery are even fewer. This gap highlights the need to examine how parents' psychological resources and caregiving behaviors shape children's adjustment after large-scale traumatic events.

Understanding the experiences of school-age children after an earthquake, being aware of their reactions, and providing assistance can be considered a crucial step (Aral, 2023). The aim of this exploratory sequential mixed-methods study was (a) to qualitatively identify parent-reported emotional and behavioral problems in children after February 6, 2023, earthquakes, parents' concerns, and coping strategies, and (b) to quantitatively test whether parental psychological resilience and parental warmth/affection predict the level of children's emotional and behavioral problems. In line with these objectives, the research questions and hypotheses have been structured as follows.

- What are the emotional and behavioral problems observed in children by parents after the earthquake?
- Which basic concerns do parents have about their children in the post-earthquake period?
- Which methods do parents use to cope with their children's emotional and behavioral problems after the earthquake?
- Are age, gender, socio-economic status, number of siblings, and relocation status related to emotional and behavioral problems in children after the earthquake?
- Do parents' psychological resilience and parental warmth predict emotional and behavioral problems in children?

Research hypotheses:

- Higher levels of parental psychological resilience predict lower levels of emotional and behavioral problems in children.
- Higher levels of parental warmth/affection predict lower levels of emotional and behavioral problems in children.

## 2 Methods

In this research, we employed an exploratory sequential mixed-methods design. The exploratory sequential design aims to collect qualitative data related to the problem, possibly develop measurement tools, and then expand these qualitative findings through quantitative data (Creswell, 2014; Creswell and Clark, 2011). A significant rationale for mixed-methods research is that the results obtained from one method can enhance the other method (Greene, 2007). In the qualitative phase of the research, a descriptive qualitative design with semi-structured interviews was used, while in the quantitative phase, a cross-sectional, correlational survey design was employed. We began our study by first collecting qualitative data. In the qualitative part of the research, we identified the emotional and behavioral problems experienced by children after the earthquake, the coping methods of parents, and their concerns about their children, based on parental views. The results obtained from the qualitative data analysis guided the quantitative design of our research and helped us expand our qualitative findings with the quantitative data we gathered. The qualitative data assisted in creating a comprehensive measurement tool to identify children's emotional and behavioral problems after the earthquake. Qualitative interviews were conducted in April–May 2023. The quantitative online survey was administered in June–July 2023, following qualitative transcription and content analysis. The interval between the two phases was approximately two months.

### 2.1 Participants

For the qualitative part of the research, the participants were determined by purposive sampling and snowball sampling methods. We used probability-based sampling to select information-rich cases (mothers of children aged 6–11 in four severely affected provinces on 6 February 2023). As many families were displaced after the disaster and a comprehensive sampling frame was unavailable, snowball sampling was employed to reach suitable caregivers through reliable networks (e.g., teachers and participating mothers) and to facilitate access to families who were difficult to reach in the post-disaster environment. The inclusion and exclusion criteria are listed below.

Inclusion criteria;

- To be a resident of one of the four provinces (Kahramanmaraş, Hatay, Adiyaman, and Malatya) where the earthquake that occurred in Türkiye on 6 February 2023 caused severe destruction and to have been in one of these provinces during the earthquake and experienced the earthquake with her child,
- Being a mother with a child aged 6–11 years,
- Living with her child after an earthquake,
- The child does not have a diagnosed psychological disorder

Exclusion criteria;

- Parents not living with their child after the earthquake
- Parents residing in provinces not severely affected by the earthquake
- Parents of children diagnosed with psychological disorders before or after the earthquake for the target age group

In the qualitative part of the study, interviews were conducted with 55 mothers who met these criteria. The main factor in conducting the interviews with mothers is that childcare is predominantly undertaken by mothers in Türkiye. Indeed, according to the results of the Turkish Statistical Institute's Family Structure Survey, it has been reported that 94.4% of women undertake childcare in household tasks (Turkey Statistical Institute Family Structure Survey (TUIK)., 2022).

The fact that mothers had the chance to observe their children more enabled the collection of more detailed and reliable data on children's emotional and behavioral reactions. Since many families were displaced after the earthquake, interviews were conducted through telephone interviews and online meetings to facilitate participation. A cross-sectional approach was adopted in the design of the quantitative part of the study. The participants of the quantitative part consisted of 398 mothers who met the inclusion criteria specified in the qualitative part of the study. The sample size for the quantitative part of the study was determined based on a priori power analysis conducted using the G^*^Power 3.1 program. For the two-tailed correlation test, α = 0.05, power 1–β = 0.80, and expected effect size *r* = 0.15 were assigned. According to (Cohen 1988), *r* = 0.15 indicates a small effect size. The analysis calculated the minimum sample size as *n* = 347; considering data losses, the study was completed with *n* = 398 participants. The *post-hoc* power analysis conducted after the study showed that the statistical power of the research was 100%. Information about the participants of the study is presented in Table 1.

**Table 1:** Demographic characteristics of the participants.

**Variables**	**Qualitative**		**Quantitative**	
	***f***	**%**	***f***	**%**
**Income status**				
Income < expenses	18	32.7	169	42.5
Income ~ expenses	31	56.3	182	45.7
Income > expenses	6	11.0	47	11.8
**Number of siblings**				
0	7	12.7	48	12.1
1	11	20.0	196	49.2
2	25	45.4	116	29.1
3 or more	12	21.8	38	9.5
**Sex of the child**				
Female	29	52.7	201	50.5
Male	26	47.3	197	49.5
**Age of the child**				
6	14	25.4	108	27.1
7	7	12.7	100	25.1
8	15	27.2	69	17.3
9	9	16.3	55	13.8
10	4	7.2	66	16.6
11	6	10.0		
**Family relocation after the earthquake**				
No	39	70.9	249	62.4
Yes	16	29.1	149	37.4
Total	55	100.0	398	100.0

### 2.2 Measures

#### 2.2.1 . Semi-structured interview form

To collect the qualitative data for the research, we used a semi-structured interview form. This form was prepared by the researchers and finalized after receiving feedback from two experts with experience in qualitative research. The interview form contains six open-ended questions. These questions are as follows:

- Did you notice any changes in your child's behavior after the earthquake? If so, what are these changes?
- Did you observe any changes in your child's emotional state after the earthquake?
- What support mechanisms (such as family support, professional help, etc.) or methods did you utilize to cope with your child's problem behaviors during the post-earthquake period?
- Did you make any changes in your daily routines or parenting approaches to manage your child's earthquake-related anxieties and problem behaviors? If so, what are these changes and what impact did they have?
- Did you have any specific concerns or fears regarding your child's safety and well-being after the earthquake?
- What kind of concerns do you have about your child's future following the earthquake?

A fundamental characteristic of semi-structured interviews is keeping the main interview questions fairly limited and expanding them with follow-up and probing questions (Rubin and Rubin, 2012). During the interviews, probing, additional explanations, and confirmation questions were posed. For example, “How did the emotional changes you mentioned reflect on your child's school performance or peer relationships?” This approach allowed for the collection of more detailed views.

#### 2.2.2 Emotional and behavioral problems questionnaire

The Emotional and Behavioral Problems Survey was developed by the researchers by compiling eighteen emotional and behavioral issues (see Table 2) identified as a result of the qualitative findings of the study. The Emotional and Behavioral Problems Questionnaire was designed as a symptom checklist (a count index) derived from the qualitative findings rather than a latent-factor scale. Each item represents a distinct emotional/behavioral problem; therefore, we focused on internal consistency (KR-20) and stability (test–retest kappa) rather than factor analytic evidence. The items in the survey are answered with “Yes (1)” or “No (0)”. As the scores obtained from the measurement tool increase, the level of experienced emotional and behavioral problems also increases. The reliability coefficient of the measurement tool, determined using KR20, has been calculated as 0.87. KR-20 is a method that measures internal consistency for measures containing dichotomous items (Ntumi et al., 2023). KR-20 value varies between 0 and 1. The closer the value is to 1, the higher the internal consistency. Values of 0.70 and above are acceptable and values of 0.90 and above indicate perfect consistency (Allen, 2017). Additionaly, since the scale statements were dichotomous items in the form of “Yes/No”, kappa consistencies were analyzed within the scope of test-retest. Emotional and Behavioral Problems Questionnaire was administered to a group of 40 participants at 3-week intervals. As a result of the calculation, kappa values ranged between 0.428 and 0.892 (*p* < 0.01) for each item. Reference intervals for interpretation of kappa values: “0.01–0.2, slight, 0.21–0.4; fair, 0.41–0.60; moderate, 0.61–0.80; substantial, and 0.81–1, almost perfect” (Landis and Koch, 1977).

**Table 2:** Emotional and behavioral problems stated in the qualitative interviews.

**Emotional and behavioral problems**	***f***
Fear of being alone	42
Constantly asking questions about the earthquake	40
Persistent worry about another earthquake occurring	40
Fear of darkness and noise	30
Inability to sleep alone	28
Constant oppositional and defiant behaviors	21
Being startled by the slightest sound and trembling	16
Difficulty falling asleep	14
Claustrophobia	11
Irritable and aggressive behaviors	10
Constantly asking about one's school/friends	9
Difficulty focusing on things	9
Postponing a task/activity in case another earthquake could occur	8
Difficulty expressing feelings	8
Constantly waking up	8
Introversion	7
Bed-wetting	7
Being worried about the future	6
Inability to do anything without one's mother or father	5
Crying spells	5
Occasional screaming	5
Thumb-sucking	3
Nail-biting	3
Hair-pulling	2
Stuttering	1

The frequencies indicate how many different participants reported the emotional or behavioral problem reported for the children.

#### 2.2.3 Brief Resilience Scale (BRS)

The Turkish validated version of the Brief Resilience Scale (BRS) developed by (Smith et al. 2008) and adapted into Turkish by (Dogan 2015) was used to measure parents' psychological resilience. It is a unidimensional measurement tool consisting of six items, based on a five-point Likert scale. The BRS is practical and suitable because it is brief, reliable, culturally adapted, and has been used in many similar studies (Canli and Yilmaz, 2024; Dinçer et al., 2025; Özer, 2024). Furthermore, it is a measurement tool that is directly appropriate for the research objective of assessing parents' ability to recover quickly from stress. Three items in the scale are reverse scored. Sample items are as follows: “*I tend to bounce back quickly after hard times*”, “*I have a hard time making it through stressful events (R)*”. In the adaptation study of the scale, Cronbach's alpha was calculated as 0.83, while in this research, it has been calculated as 0.71. A Cronbach's alpha value of at least 0.70 is accepted as the practical threshold value (Nunnally and Bernstein, 1994). As the scores obtained from the BRS scale increase, psychological resilience also increases.

#### 2.2.4 Parental Acceptance-Rejection Questionnaire (PARQ)

To measure parental warmth, the “warmth/affection” subdimension of the short form of the PARQ, developed by (Rohner 2005), was used. This questionnaire was adapted into Turkish by (Renklibay 2017). The scale is valid and reliable for measuring parental warmth, and because it is particularly short, it allows data to be collected without placing an additional burden on participants. The warmth/affection subdimension of the questionnaire, which consists of twenty-four items, is made up of eight items. Sample items are as follows: “*I say nice things about my child*”, “*I make it easy for my child to confide in me*”. In the adaptation study of the scale, Cronbach's alpha was calculated as 0.84, while in this research, it has been calculated as 0.70. An increase in the score obtained from the questionnaire means that the affection/warmth of the parent toward the child increases.

### 2.3 Qualitative data collection and analysis

The qualitative data for the research began to be collected 2 months after the earthquakes. The University Ethics Committee approved the study. For participant selection, the snowball sampling method was chosen. In this method, researchers initially made effective use of their social networks. One of the researchers, X, aimed to reach potential participant families through teachers residing in and assigned to the earthquake-affected areas. Another researcher, Y, contacted dormitories hosting earthquake victims in different provinces. During the selection of participants, verbal and written consent processes were carefully implemented, and individuals who met the inclusion criteria were included in the research process. In order to broaden the scope of the study, we leveraged recommendations from parents who had previously participated in the study to identify new potential participants. This method greatly diversified the participant pool for the research. Before conducting the interviews, the participants were briefed about the recording process, and their consent was secured, either orally or in written form. The recordings were made to ensure the collected data's accuracy and the research methods' transparency. The interviews typically lasted around 25 min on average. This process was integral to upholding the ethical standards and systematic approach of the qualitative data collection methods employed in the study.

Before the qualitative data was analyzed, it was transcribed and carefully recorded, with a detailed check for accuracy following the transcription. Transcripts were sent to the participants via e-mail and their accuracy was confirmed. Content analysis technique was utilized for the analysis of the data. Content analysis aims to reach concepts and relationships that can explain the data, thereby uncovering unnoticed themes and concepts. Additionally, in content analysis, each research question can be considered a theme for the analysis (Yıldırım and Şimşek, 2011). Qualitative data analysis was conducted to answer the first three research questions. In this context, these three research questions were accepted as themes. Consequently, themes such as emotional and behavioral problems in children post-earthquake, coping strategies of parents, and parents' anxieties and fears were identified. In the next stage, two researchers read the interview texts separately and placed appropriate codes under these themes. Inter-coder agreement percentage was calculated to determine the agreement between the coding of the two researchers. As a result of this calculation, the percentage of agreement was calculated as 86.4%. After this process, the whole research team came together to discuss the appropriateness of the codes classified under each theme. For the codes that the coders could not reach a consensus, a decision was made in line with the third researcher's opinion. In the last stage, the relationships between the codes were evaluated and sub-themes were formed. The results of the content analysis were reported with frequency analyses or visual presentations of the relevant themes according to the nature of the findings.

### 2.4 Quantitative data collection and analysis

The quantitative data collection for the research commenced after the gathering and analyzing the qualitative data. A period of approximately 2 months passed between the collection of qualitative and quantitative data. The data were collected through an online form. Parents who met the inclusion criteria specified in the qualitative part of the study were included in the quantitative part. Participants were reached using the snowball sampling method. Initially, parents who participated in the qualitative dimension of the research were approached. After completing the online form, they were asked to share it with families in their networks affected by the earthquake. In addition, while collecting quantitative data, if a participant had more than one child in the target age range, the participant was asked to provide data about the younger child. Thus, nestedness was prevented in data analysis.

The analysis of the quantitative data was conducted using the SPSS program. Initially, it was analyzed whether there were missing or incorrect data and it was found that there were no missing or incorrect data. In the next stage, Kolmogorov-Smirnov test was used to determine if the data were normally distributed, and it was found that the data were not normally distributed (*p* < 0.05). However, the data are normally distributed if the sample size is greater than fifty and the Skewness and Kurtosis values are between −1.5 and +1.5 (Tabachnick and Fidell, 2013). In this study, the skewness and kurtosis values of the scales used were between −1.5 and +1.5. Hence, it was accepted that the data were normally distributed. Furthermore, total scores obtained from Likert-type scales converge to continuous variables in large samples. Therefore, the use of parametric tests is considered appropriate. Based on this result, an independent groups t-test was used for binary groups, one-way analysis of variance (ANOVA) for groups of three or more, Pearson correlation analysis to determine the relationship between scales, and hierarchical regression analysis for prediction analysis. A prerequisite of hierarchical linear regression analysis is the absence of multicollinearity among variables. This context examined tolerance and variance inflation factor (VIF). It was determined that the tolerance values were higher than zero, and the VIF values were less than 10. The tolerance values far from zero and VIF values less than 10 indicate no multicollinearity problem (Field, 2009). The research met the assumptions and conducted a hierarchical linear regression analysis. A theoretical sequence was followed in establishing the hierarchical regression model. In the first block of the model, demographic variables (household income and number of siblings) that affect children's emotional and behavioral problems were included in the model as control variables. Since these variables are categorical, a reference category was determined before including them in the model, and dummy variables were created for the other categories. This allowed us to estimate the unique predictive effects of parental psychological resilience and parental warmth/affection beyond basic demographic/contextual factors. In the second block, parental psychological resilience and parental warmth were included because they were defined as psychological variables related to children's emotional and behavioral characteristics. Effect sizes (partial η^2^) and 95% confidence intervals were reported in all analyses. Finally, (Cohen 1988) effect size values for variance analyses, defined as small (η^2^ = 0.01), medium (η^2^ = 0.06), and large (η^2^ = 0.14), were considered in interpreting the effect size.

## 3 Results

### 3.1 Qualitative analysis results

#### 3.1.1 Emotional and behavioral problems observed by parents

We begin our findings with those obtained from the analysis of qualitative data. The emotional and behavioral problems experienced by children after the earthquake, as per the parents' views, are presented in frequency in Table 2. The most frequently observed emotional and behavioral problems in children identified by parents include fear of being alone (*f* = 42), constantly asking questions about the earthquake (*f* = *40*), persistent worry about another earthquake occurring (*f* = 40), and fear of darkness and noise (*f* = 30).

The least reported problems by parents tend to be more behavioral in nature. Some of these issues include stuttering (*f* = 1), hair-pulling (*f* = 2), nail-biting (*f* = 3), and thumb-sucking (*f* = 3). Additionally, problems related to sleep, social issues, and difficulties with emotion and anger control have also been mentioned by parents.

#### 3.1.2 Fears and anxieties of parents about their children

Another qualitative research finding focused on parents' anxieties regarding their children (Table 3). Only seven parents reported not having any fear or anxiety about their children. However, other parents expressed their concerns and fears about their children and mentioned their requests to authorities, such as being patient, constructing safe living areas, and being particularly involved in psychosocial support activities. Parents frequently reported worries about the psychological problems their children experienced after the earthquake and how these issues would affect their lives in the long term. They also expressed concern about situations such as children being separated from their friends, rooms, and toys after the earthquake, which could trigger loneliness and separation anxiety, and how their children would overcome these situations. One participant expressed their views on the long-term effects: “*What the children have experienced is not easy and will definitely cause them psychological problems in the future. Children have strong memories. I don't think they will easily forget what they've been through*.” Another parent shared: “*This earthquake caused great destruction for him, and being a very emotional child, I think he will never be able to erase it from his memory*.” Parents reported living with the fear of losing their children. They mentioned that ongoing aftershocks and security concerns after the earthquake triggered this fear, causing anxiety and worry in daily life. A parent stated: “*I fear that my child will be trapped under the rubble if there is another earthquake*.” Parents also expressed concerns about their children's difficulties adapting to school after the earthquake. Moving to a new city or starting a new school could affect the children's adaptation process. One participant shared: “*He doesn't want to study because his fear is unending, and I don't believe he will be as good in his studies as before*.” Lastly, parents frequently reported anxieties about their children's futures. They expressed concerns about ensuring safety and establishing a normal routine for the future. Parents often emphasized the difficulties of adjusting to life in a new city or a new home. They also reported being unable to make future plans due to the uncertainty they were experiencing. One parent expressed: “*I can't make promises about the future to them because we don't know what will happen next and what our lives will be like*.” Another parent stated: “*I don't know where and how to start since our entire routine has been disrupted. We are in a big uncertainty regarding the future*.”

**Table 3:** Parents' anxieties/fears about their children and coping strategies.

**Themes related to parents' fears and concerns**	**Themes of coping strategies used by parents**
•Fears about long-term effects on children •Education-related concerns •Adjustment to the environment •Fear of loss •Future anxiety	•Spending time together •Showing warmth and affection •Informing children about the earthquake •Keeping children away from the media •Staying strong as parents and promoting hope •Promoting return to normal life •Spiritual support

#### 3.1.3 Parents' strategies for coping with emotional and behavioral problems in children

Another finding from our qualitative research pertains to the strategies parents use to cope with their children's emotional and behavioral problems (Table 3). Of the participating parents, only ten reported receiving psychosocial support, while the others mentioned trying to overcome these situations using their own methods. Parents frequently reported spending quality time together and showing warmth to their children as strategies for coping with their children's emotional and behavioral issues. Some parents also mentioned providing information to their children about earthquakes, thus helping them accept it as a normal part of life. Many parents tried to keep their children away from the media as a protective strategy. Some parents noted that even the media images affected them. One parent described the situation: “*During the earthquake, we stayed home and didn't involve the children in earthquake conversations or news. Otherwise, they would experience it more like everyone else*.” An important strategy highlighted by parents was to remain strong and encourage their children's hope for the future. One participant expressed their views on this theme: “*I told them to be patient, that everyone is in the same situation, things will get better, and we will eventually get back to our normal routine*.” Another strategy reported by parents was trying to maintain their previous routines and life order. Finally, regarding spiritual support, parents shared that they prayed with their children and helped them. One parent shared: “*We pray with my children that there will be no more earthquakes and imagine going to a country far from the earthquake zone, trying to help them forget this way*.”

### 3.2 Quantitative analysis results

The quantitative data analysis resulted in descriptive statistics for the Emotional and Behavioral Problems Questionnaire, provided in Table 4. The children participating in the study most frequently experienced problems such as “fear of being alone” (83.9%), “fear of darkness and noise” (76.9%), “constantly asking questions about the earthquake” (73.4%), “persistent worry about another earthquake occurring” (70.9%), and “inability to sleep alone” (70.4%). The least experienced emotional and behavioral problems by the children were “becoming introverted” (22.6%), “frequently waking up from sleep” (32.2%), “difficulty in expressing emotions” (36.9%), “worrying about the future” (43.7%), and “becoming unable to do anything without a parent” (45.5%).

**Table 4:** Emotional and behavioral problems questionnaire.

**Emotional and behavioral problems**	**Present**	**Absent**
Fear of being alone	334 (83.9%)	64 (16.1%)
Fear of darkness and noise	306 (76.9%)	92 (23.1%)
Constantly asking questions about the earthquake	292 (73.4%)	106 (26.6%)
Persistent worry about another earthquake occurring	282 (70.9%)	116 (29.1%)
Inability to sleep alone	280 (70.4%)	118 (29.6%)
Constantly asking about one's school/friends	261 (65.6%)	137 (34.4%)
Claustrophobia	237 (59.5%)	161 (40.5%)
Irritable and aggressive behaviors	234 (58.8%)	164 (41.2%)
Constant oppositional and defiant behaviors	229 (57.5%)	169 (42.5%)
Being startled by the slightest sound and trembling	224 (56.3%)	174 (43.7%)
Difficulty focusing on things	219 (55.0%)	179 (45.0%)
Difficulty falling asleep	200 (50.3%)	198 (49.7%)
Postponing a task/activity in case another earthquake could occur	188 (47.2%)	210 (52.8%)
Becoming unable to do anything without a parent	181 (45.5%)	217 (54.5%)
Worrying about the future	174 (43.7%)	224 (56.3%)
Difficulty in expressing emotions	147 (36.9%)	251 (63.1%)
Frequently waking up from sleep	128 (32.2%)	270 (67.8%)
Becoming introverted	90 (22.6%)	308 (77.4%)

The results of the mean, standard deviation, and correlation analysis for the variables are presented in Table 5. The results of the correlation analysis show that there is a significant negative relationship between emotional and behavioral problems in children reported by parents and the psychological resilience of parents [*r*_(398)_= −0.332; *p* < 0.001]. However, the relationship between emotional and behavioral problems in children reported by parents and parental warmth [*r*_(398)_= −0.015; *p* > 0.05] and between psychological resilience and parental warmth [*r*_(398)_= 0.057; *p* > 0.05] is not significant.

**Table 5:** Descriptive statistics and correlation analysis.

	**Mean**	**SD**	**1**	**2**	**3**
(1) Emotional and behavioral problems questionnaire	10.06	4.736	1		
(2) Psychological resilience	18.42	3.822	−0.332^**^	1	
(3) Parental warmth/affection	30.74	1.698	−0.015	0.057	1

^**^p < 0.001.

The comparison of the mean scores related to emotional and behavioral problems reported by parents for children in terms of demographic characteristics is presented in Table 6. The results of the analysis show that the level of emotional and behavioral problems reported by parents for children differed significantly depending on the income level of the family (*F*_(2 − 395)_= 6.645; *p* < 0.05) and the number of siblings (*F*_(3 − 394)_= 3.333; *p* < 0.05). Accordingly, parents with lower income levels reported more problems than those with higher income levels, and parents with two children reported more problems than those with one child. However, the mean scores of emotional and behavioral problems reported by the parents did not differ significantly according to the child's gender (*t*_(398)_= 0.377; *p* > 0.05), age (*F*_(4 − 393)_= 0.750; *p* > 0.05) and post-earthquake relocation status (*t*_(398)_= 0.125; *p* > 0.05).

**Table 6:** Differences in emotional and behavioral problems based on demographic characteristics.

**Variables**	**n**	**Mean**	**SD**	**Test**	**Sig**.	**Effect size**
**Income**						
(1) Income < expenses	169	11.04	4.918	*F* = 6.645	*p* < 0.001	η^2^=0.033
(2) Income ~ expenses	182	9.47	4.531		1 > 2	
(3) Income > expenses	47	8.87	4.261		1 > 3	
**Number of siblings**						
(1) 0	48	8.65	4.329	*F* = 3.333	*p* = 0.020	η^2^=0.025
(2) 1	196	10.75	4.764		2 > 1	
(3) 2	116	9.58	4.668			
(4) 3 or more	38	9.79	4.855			
**Sex**						
Female	201	10.15	4.516	*t* = 0.377	*p* = 0.706	
Male	197	9.97	4.960			
**Age of the child**						
(1) 6	108	9.44	4.962	*F* = 0.750	*p* = 0.558	
(2) 7	100	10.40	4.228			
(3) 8	69	10.04	4.983			
(4) 9	55	10.15	4.648			
(5) 10	66	10.53	4.927			
**Family relocation after the earthquake**						
No	249	10.09	4.673	*t* = 0.125	*p* = 0.900	
Yes	149	10.03	4.855			

t= Independent-samples t-test; F= one-way analysis of variance (ANOVA).

The results of the hierarchical regression analysis are presented in Table 7. Firstly, the control variables of income and number of siblings were added to the model (see model 1). In the next step, the main independent variables of parental warmth and psychological resilience were added (see model 2). In Model 1, the analysis results show that the control variable income predicts the emotional and behavioral problems in children reported by parents negatively and significantly (β = −0.175^**^). In contrast, the contribution of the number of siblings to the model is insignificant (β = −0.013). This finding indicates that higher-income families report lower emotional and behavioral problems for their children. In Model 2, psychological resilience and parental warmth were added to the model as the main independent variables in addition to income and number of siblings. The results of the analysis revealed that parental psychological resilience significantly predicted emotional and behavioral problems in children reported by parents (β = −0.312^**^), the significant contribution of income to the model continued (β = −0.123^*^), and the contribution of parental warmth to the model was insignificant (β = 0.002). In other words, higher parental resilience and family income are associated with lower emotional and behavioral problems in children parents report. In addition, the final model (model 2) can predict approximately 12% of the emotional and behavioral problems in children reported by parents.

**Table 7:** Hierarchical regression analysis.

**Independent variables**	**Model 1 (95%CI)**	**Model 2 (95%CI)**
Income	−0.175^**^ (−0.272/−0.078)	−0.123^*^ (−0.217/−0.029)
Number of siblings	−0.013 (−0.111/0.084)	−0.000 (−0.094/0.093)
Psychological resilience		−0.312^**^ (−0.407/−0.218)
Parental warmth/affection		0.002 (−0.091/0.096)
*F*	6.237^*^	14.074^**^
*R*	0.175	0.354
$R_{\left(\text{Adjusted}\right)}^{2}$	0.026	0.116
Δ*R*^2^		0.090^**^

^*^p < 0.05; ^**^p < 0.001; ΔR^2^ = R Square Change; standardized regression coefficients are presented.

## 4 Discussion

This research examined the emotional and behavioral problems occurring in children after an earthquake, based on parental experiences, using a mixed-method (exploratory sequential design) approach. We also compared emotional and behavioral problems in children in terms of specific demographic variables and determined whether parental resilience and parental warmth predicted emotional and behavioral problems in children as reported by parents.

Our qualitative data analysis provided a comprehensive framework for understanding emotional problems and consequent behavioral issues that can occur in children following an earthquake. The most frequently reported problems by parents are emotional issues such as fear of loneliness, constantly asking questions about the earthquake, persistent worry about future earthquakes, fear of darkness, and inability to sleep alone. These are emotional issues related to stress, anxiety, and insecurity triggered by the earthquake. Additionally, parents have reported numerous behavioral problems, including stuttering, hair-pulling, thumb-sucking, and nail-biting. Moreover, parents identified issues such as sleep disturbances, social challenges, and problems with anger management in their children. Our quantitative analysis strongly supports the prevalence of emotional and behavioral difficulties in children identified through our qualitative analysis. Typically, children display psychological reactions such as PTSD (Pynoos et al., 1993) and depression (Becker-Blease et al., 2010), along with experiences of fear, sadness, and anxiety (Niman et al., 2023) following such events. These emotional struggles often result in a range of behavioral issues, such as aggressiveness, bed-wetting, and excessive crying (Madrid et al., 2006). Our research corroborates existing studies on children's responses in disaster situations, shedding light on their emotional and behavioral responses. This comprehensive understanding is crucial for better grasping children's reactions and developing appropriate support mechanisms. In addition, it is recommended that caregivers of children exposed to disasters should carefully monitor the emotional and behavioral reactions of these children and ask for expert support if necessary.

The outcomes of our investigation reveal that parents have implemented various methods to address their children's emotional and behavioral problems. These strategies encompass spending meaningful time together, demonstrating affection and warmth, educating children about the earthquake, limiting their exposure to media, maintaining a strong and hopeful demeanor as parents, encouraging a return to regular routines, and offering spiritual guidance. These methods can meet children's social and emotional needs and help them cope with their fears. Indeed, children feel secure with cues from their parents (Madrid et al., 2006), and the emotional closeness and support they provide can significantly impact children. Numerous empirical studies demonstrate the relationship between the mental health of parents post-disaster and that of their children (North et al., 2018; Wickrama and Kaspar, 2007). However, there is insufficient evidence on which attitudes and behaviors of parents can predict emotional and behavioral problems in children. Parents frequently emphasized some themes to cope with emotional and behavioral problems in children after the earthquake. In particular, warm/compassionate behavior toward children and staying strong and encouraging hope as an indicator of psychological resilience were prominent coping methods. As part of the exploratory mixed-method research process, we hypothesized that these two factors might predict emotional and behavioral problems in children.

Our quantitative data analyses show that parental resilience is a significant negative predictor of emotional and behavioral problems reported by parents for children. In contrast, parental warmth is not a significant predictor. Various factors related to relationships with other individuals play a role in determining children's reactions to disasters; among these, parents' reactions and coping styles are the main ones (Cadamuro et al., 2021). According to (Aral 2023), children perceive the adults around them as fearless and able to overcome all kinds of difficulties; however, the emotional reactions of adults in situations such as earthquakes can cause serious insecurity in children. Parents with high psychological resilience may better adapt to stressful situations, develop problem-solving skills, and have more positive expectations for the future. Psychological resilience is likely a protective factor for parents' mental health.

Children are often influenced by their parents' emotional reactions in understanding and coping with the effects of disaster. Children feel safe when parents provide their children with love, trust, and a calm demeanor (Prinstein et al., 1996). In addition to being the mainstay of interventions for children, parents play a critical role in linking children to community resources. Our findings indicate that parental warmth alone is not a significant predictor of emotional and behavioral problems. This suggests that, while love and calm communication are valuable, they may not be sufficient to alleviate children's post-disaster distress without parental resilience. Interventions implemented at the family and community level are based on the underlying assumption that recovery stems from the restorative nature of interpersonal relationships. The resilience exhibited by individuals is often fueled by the care and encouragement they receive from others, as well as their own motivation and intention to lighten the burden of others (Margolin et al., 2010). Consistent with this perspective, our findings confirmed that parents' psychological resilience significantly predicted children's emotional and behavioral problems in a negative direction, confirming that resilience is a fundamental protective factor in post-disaster family systems. Parents with low psychological resilience and adjustment difficulties are likely to be unable to provide their children with the support they need after disasters and such parents may model trauma-related symptoms to their children (Weissbecker et al., 2008). Previous research underlines the importance of parents as a primary source of social support (Norris et al., 2002; Ronan et al., 2008). However, it does not say much about how parents approach their children and what kind of support they offer. In this context, the result of our study underlines that there is no significant relationship between parents' affection/warmth toward their children and emotional and behavioral reactions in children after the earthquake. In a study conducted on adolescents exposed to tsunamis (Wickrama and Kaspar, 2007), positive mother-child relationships were found to have a compensatory effect on both depressive and PTSD symptoms of adolescents. These results highlight the importance of understanding supportive attitudes and behaviors in the parent-child relationship after a disaster. Our results emphasize that parents' psychological resilience is related to emotional and behavioral problems in children. In addition, our results provide evidence for approaches that advocate for collective resilience in traumatic events such as earthquakes and promote community healing as a whole (Bonanno et al., 2010; Cadamuro et al., 2021). Promoting parents' psychological resilience can be seen as a disaster risk reduction strategy for themselves and their children. In the intervention programs to be developed, integrated models that will include their social environment instead of approaches to treat only the distress and disorders in children may be a more effective way and support collective recovery.

The research results indicate that parents have numerous concerns and fears regarding their children. Long-term negative effects of the earthquake on children, fear of losing a child, educational concerns, issues with environmental adaptation, and anxieties about the future are among the worries parents report. This also shows that parents' concerns extend beyond the child's safety to the child's developmental and social future. These concerns can be a secondary source of stress for parents in the wake of a disaster and may exacerbate their psychological problems. Additionally, these findings point to areas of focus for social intervention programs. For parents, worries about their children, in addition to experiencing the disaster, are stressors that perpetuate the negative effects of the earthquake (Lock et al., 2012). A study by (North et al. 2018) on how family members affect each other's mental health after a disaster found that the level of PTSD in parents is related to their children's distress. Furthermore, a study by (Wickrama and Kaspar 2007) found that high levels of depressive symptoms among mothers after a tsunami had detrimental effects on adolescent mental health. These anxieties and fears of parents can affect the recovery process of children.

In our quantitative data analysis, we compared emotional and behavioral problems in children based on income status, number of siblings, gender, age of the child, and relocation status. The analysis determined that family income and the number of siblings impact children's emotional and behavioral problems. The child's gender, age, and city change were not found to be related to these problems. Income status is key to meeting many basic needs, such as food, clothing, and shelter after a disaster. Failure to meet these needs functions as a source of stress (Lock et al., 2012). Economic insufficiencies increase vulnerability for both parents and children (Peek, 2008). Therefore, our quantitative results empirically support these theoretical claims and demonstrate that post-disaster psychological well-being is closely related to material stability. In the aftermath of disasters, individuals with better economic status may have the opportunity to migrate to places with better conditions, meet the shelter, education, and psychosocial support needs of children, and provide additional support. Poor individuals, on the other hand, try to continue their lives in collective shelter centers, tents, or under more difficult conditions. In addition, previous studies have found that low-income families have problems accessing shelter, food, and health services after a disaster (Brunson, 2017), and mental health problems are more common in children in these families (Pfefferbaum et al., 2015). Our results also show that children in socio-economically disadvantaged families are more vulnerable to disasters. In line with these findings, it can be said that it is critical to structure post-disaster intervention programs in a way that strengthens the support systems of families with low socio-economic status. In this context, it is recommended to prioritize the shelter, education and psychosocial support needs of children and to make arrangements to alleviate families' economic difficulties.

Our findings suggest that parents with one child report fewer emotional and behavioral problems with their children than those with two children. Children without siblings may receive more support from their parents as a resource. However, studies also report that more children in a family are associated with fewer behavioral problems, depending on the support provided by siblings (North et al., 2018). (Xu et al. 2013) linked older children's increased responsibilities with higher stress levels. However, emotional and behavioral problems in other siblings may also lead to more negative reactions. Thus, having a sibling may not always be a source of support. These results suggest that intervention programs should be planned to meet the different needs of both multi-child and single-child families by considering sibling dynamics and the distribution of resources within the family. Therefore, it is thought to be important to develop flexible and inclusive strategies that holistically address the age and responsibility level of the child, the quality of sibling support, and the stress sources experienced within the family in post-disaster interventions. Research findings generally show that girls report more problems than boys (Karakaya et al., 2004; Pynoos et al., 1993; Rezayat et al., 2020). However, in a study conducted by (Bulut 2009), which supported our results, no difference was found in terms of gender. In the study conducted by (Erkan 2010) comparing the behavioral/emotional problems of preschool children with and without earthquake experience, it was found that the main effect of gender on the problem behaviors of the children in the sample was significant regardless of the earthquake experience and that boys had somatic problems and girls had more attention problems. In this context, our findings confirm that gender may not be a strong determinant of post-disaster adaptation and suggest that differences between boys and girls may be related to contextual factors such as exposure intensity or parents' coping styles rather than gender. As a result, gender comparisons regarding children's problems after disasters give quite mixed results. In this context, it can be said that more research is needed, and different variables should be considered in gender assessment. Some studies report that young children are more vulnerable in terms of emotional and behavioral problems (Lieber, 2017), but our results show that age does not affect problems in children. However, children's behavioral and emotional reactions may differ according to age. Our findings show no relationship between post-disaster relocation and problem behaviors reported by parents for children. Previous studies (Karabulut and Bekler, 2019; Najarian et al., 1996; Wickrama and Kaspar, 2007) reported that post-disaster displacement is associated with psychological problems in children. In addition, (Scannell et al. 2016) stated that place attachment is important for children and youth's disaster preparedness, experiences, recovery, and resilience. In this context, the authors claimed that displacement after a disaster can cause significant emotional distress among children and youth. Our results do not support this view. This suggests that there may not be a uniform relationship between post-disaster displacement and problems in children. Many factors, such as post-disaster support, family income, individual differences, and family dynamics, may be determinants of emotional and behavioral problems in children.

Overall, the mixed-method results converge in showing that school-age children experienced prominent post-earthquake emotional difficulties (e.g., fear, persistent earthquake-related worry) that were frequently accompanied by behavioral manifestations. Importantly, the survey prevalence pattern mirrors the problems most commonly emphasized by parents in the interviews, strengthening the credibility of the qualitative framework. The qualitative phase further indicated that parents actively mobilized coping resources, frequently emphasizing warmth/affection, maintaining routines, limiting media exposure, and “staying strong” to reassure their children. The quantitative phase refined these insights by showing that parental psychological resilience (and family income) was a significant protective predictor of parent-reported child emotional and behavioral problems, whereas general parental warmth/affection was not a significant predictor in the regression model. This divergence suggests that warmth may represent a widely endorsed coping response with limited between-parent variability in this sample and/or may operate indirectly (e.g., as a moderator under high distress or through other family processes) rather than as a direct predictor. Taken together, the integrated evidence highlights the importance of interventions that strengthen parents' resilience and broader family resources while also supporting parent–child relational practices identified in the qualitative findings.

## 5 Limitations and future directions

Although the purposive and snowball sampling methods used in this study facilitated reaching families who relocated after the earthquake, the fact that the participants were mostly selected on a voluntary basis limits the generalizability of the findings. In addition, the fact that the data is based only on mothers' self-reports carries the risk that children's problems cannot be verified from other sources, such as teacher reports, fathers or expert observations. The cross-sectional design used in the study does not allow examining changes over time and long-term effects. Additionally, the Emotional and Behavioral Problems Questionnaire, which we developed based on qualitative research findings, can be evaluated more comprehensively from a psychometric perspective in future studies. In this context, its structural validity can be tested using confirmatory factor analysis (CFA) in different samples. Furthermore, criterion validity can be examined by comparing it with standardized measurement tools that have proven validity and reliability in assessing behavioral problems in children.

Parents' indirect experience of distress by observing their children's distress may either increase or decrease their own psychological resilience (Cadamuro et al., 2021). Another important limitation of the study is not taking into account such reciprocal effects. It is suggested that future research should focus on the factors that promote parents' psychological resilience after a disaster. This aligns with community-based strategies that move away from an individual-based resilience-building approach (Höfler, 2014). In addition, it is known that many factors, such as the social support provided after the disaster, the degree of damage caused by the natural disaster, and the child's medical background, are important factors in determining the effects of the disaster on children. However, the fact that these factors were not examined in detail within the study limits the scope of the results (Kurt and Gülbahçe, 2019). It is recommended that future studies should consider the effects of disasters on children from a broader perspective by taking these variables into account.

From a community psychology perspective, emotional and behavioral problems seen in children after the earthquake are not only related to individual and family factors but also to empowerment, access to resources, and social support mechanisms at the community level (Morgado, 2020). In this context, future studies should examine the structure, accessibility, and effectiveness of social support networks, especially after disasters, in a way that contributes to the community psychology literature. Research focusing on the role of social ties and solidarity in children's recovery processes can guide the design of individual and community-based interventions. In addition, studies assessing whether intervention programs that aim to increase resilience at the community level are effective in reducing children's emotional and behavioral problems are important. Finally, it is emphasized that integrated models that include community and environmental factors should be developed beyond individual-level interventions. In this context, a comprehensive examination of the multifaceted interactions between children's distress, parental resilience, and socio-cultural factors may contribute to understanding post-disaster recovery processes.

## 6 Conclusion

In this research, the emotional and behavioral problems that emerge in children following an earthquake and the family-function related factors that could predict these problems have been examined through a mixed-method research approach. The study has revealed significant findings related to the complex emotional and behavioral issues in children post-earthquake. In line with the purpose of the study and the research questions, the qualitative phase revealed parents' observations regarding their children's emotional/behavioral problems, parental anxieties, and coping strategies. Afterwards, these themes were tested in the quantitative phase within the exploratory sequential design framework through demographic variables and parental variables. It has also been concluded that the psychological resilience of parents can significantly predict the emotional and behavioral problems in children, while the warmth/affection shown by parents toward their children does not predict these problems. Importantly, the problems most frequently expressed in the qualitative findings (e.g., fear of being alone, fear of the dark/sounds, repetitive questions about earthquakes, and difficulties related to sleep) were also among the most frequently reported problems in the quantitative assessment. This situation showed that the qualitative and quantitative findings overlapped. The research results provide important information related to factors associated with parents in preventing these emotional and behavioral problems in children after an earthquake. These findings can be considered in the development of post-disaster intervention and support programs.

Lastly, the research findings have unveiled the relationship between children's social and emotional behaviors and demographic variables such as family income and number of siblings. These findings indicate that variables related to resources and family structure (household income and number of siblings) are associated with the emotional and behavioral problems reported by parents regarding their children. However, the children's age, gender, and whether they had moved or not were not found to be associated in this sample. These results indicate that families with lower socio-economic status are more vulnerable to the destructive effects of disasters, and that family structure is an important variable. Research findings emphasize that post-disaster interventions should aim to both strengthen parents' coping capacity and reduce the resource constraints experienced by families. It is recommended that these factors be considered with a holistic approach in post-disaster interventions.

## Data Availability

The raw data supporting the conclusions of this article will be made available by the authors, without undue reservation.
